# Moving beyond algorithmic accuracy to improving user interaction with clinical AI

**DOI:** 10.1371/journal.pdig.0000222

**Published:** 2023-03-22

**Authors:** Shlomo Berkovsky, Enrico Coiera

**Affiliations:** Centre for Health Informatics, Australian Institute of Health Innovation, Macquarie University, Sydney, Australia; University of Pittsburgh, UNITED STATES

Clinical AI has made enormous progress recently with some algorithms performing comparably to humans for a wide range of diagnostic and therapeutic applications [1,2]. Despite this progress, evaluating AI performance until recently has typically focussed on perfecting the accuracy of the AI. That is, developers and practitioners pay attention to computational metrics such as sensitivity, specificity, and AUC as the main performance indicators. In response, there has been a push to shift the focus on more meaningful clinical outcomes, as excellent technical performance on a task might not offer tangible real-world benefits.

Understanding how best to fit AI into clinical workflows and how humans and AI best come together to deliver best clinical outcomes–without interfering with the clinicians’ work [3], disrupting clinical protocols [4], or introducing new risks [5]–represents the next research frontier. There is a chain of events that must occur between use of an AI and its real-world impact [6]. We argue that *user interaction* is a critical missing links between computational performance and clinical outcomes [7]. How often an AI is used and how well users can harness its benefits are critical to converting the tremendous potential of AI into clinical reality.

User interaction in this context refers to the harmonious and complementing fit between a human and AI, and the roles and tasks that each side takes [8]. In other words, our attention shifts from the AI *per se* to its affordances to support a collaborative human-AI team. There is no canonical design for such interaction, as the degree to which an AI is subordinate to human decision-making, or is allowed to operate autonomously, is task- and context-dependent [9]. An AI can take over mundane and labour-intensive tasks, such as image processing, signal analysis, clinical text processing, or biomarker identification. On the other hand, the interpretation of information surfaced by the AI, as well as diagnostic and therapeutic decisions, will likely be left with a human clinician.

In this paper, we turn our attention to user interaction factors that may hinder the translation of clinical AI, no matter how accurate it is. For this, we treat AI as a black box, to focus just on *how human clinicians can best harness it*. Thus, we leave explainablity beyond the scope of discussion because it affects the design of AI. While a range of interaction factors could be considered, the following three seem particularly important because of their potential to improve the adoption and deployment of clinical AI and, consequently, improve clinical care.

1. **Minimal-demand interaction**. Cognitive load on clinicians is often high, partly due to the limited usabilty of digital technologies like electronic records, which consume time and attention that would otherwise be allocated to patients [10]. If not designed properly, we can realistically expect clinician interaction with AI to further aggravate this problem. Thus, clinical AI needs not just to be accurate, but also *usable*. Getting there will likely require emphasis on features like intuitive and easy-to-learn interfaces, unambiguous information presentation, easy ways to navigate various interfaces and options, high user satisfaction and experience, adaptability of the interface and service, etc. The lower the cognitive resource required to interact with clinical AI, the higher the chance for its effective use.
2. **Innovative interaction paradigms**. Stepping beyond keyboard-and-screen interaction, one can imagine more convenient interaction paradigms with AI fuelled by sensing technologies and ambient computing. *Natural* interaction–for instance, using covert carefully designed voice prompts, gestures, or gaze–may facilitate hands-free information access, not disrupting the flow of patient conversation and treatment [11]. Gaze-driven interaction seems particularly compelling, as this will pave the way for hand-free and seamless clinician support. Combined with head-mounted displays, these are intriguing means to streamline interaction and improve consultation. Many clinicians may prefer to access AI through existing clinical system like electronic health records. Hence, better integration of such innovative interaction paradigms into the current clinical protocols is an essential step towards their adoption.
3. **Intelligent interaction**. Adding intelligence to human-AI interaction offers an alternative path to enhance AI use. This may upgrade the current clinician-driven interaction into a mixed-initiative interaction, where some AI tasks are performed proactively, without being explicitly requested by the clinician [12]. Alerts are a common example and, in more advanced scenarios, one can imagine adaptive information presentation, prioritisation of electronic communication, contextual evidence search, or automated population of medical records. If implemented gradually, in a way that keeps the clinician in control, such intelligent interactions can reduce the time and effort required to navigate vast volumes of information accessible to medical AI and streamline the use of the AI. The imperative for rigorously evaluating such proactiveness is evident, and needs to happen not just at initial certification, but also as systems are deployed.

We believe that getting the *interaction* between clinicians and AI right is the next challenge for healthcare AI developers. There is a huge and rich literature to tap into, but the peculiarities and complexities of the clinical workplace likely mean that there will be no one-size-fits-all solution. Rather a principled and context sensitive approach, taking into consideration the needs and view-points of various stakeholders, is needed. This has been done in a bottom-up manner for some clinical tasks [13] and specialisations [14], while a holistic centralised attempt for a systemic change may be more appropriate. Critically, we must recognise that beyond getting the interaction right, our goal is not to do so at the expense of other tasks clinicians perform, so to maximise practical benefits of the AI and improve the quality of patient care.
